# Pre-training Skeletal Muscle Fiber Size and Predominant Fiber Type Best Predict Hypertrophic Responses to 6 Weeks of Resistance Training in Previously Trained Young Men

**DOI:** 10.3389/fphys.2019.00297

**Published:** 2019-03-26

**Authors:** Cody T. Haun, Christopher G. Vann, C. Brooks Mobley, Shelby C. Osburn, Petey W. Mumford, Paul A. Roberson, Matthew A. Romero, Carlton D. Fox, Hailey A. Parry, Andreas N. Kavazis, Jordan R. Moon, Kaelin C. Young, Michael D. Roberts

**Affiliations:** ^1^School of Kinesiology, Auburn University, Auburn, AL, United States; ^2^Department of Exercise Science, LaGrange College, LaGrange, GA, United States; ^3^Impedimed, Inc., Carlsbad, CA, United States; ^4^Department of Cell Biology and Physiology, Edward Via College of Osteopathic Medicine – Auburn Campus, Auburn, AL, United States

**Keywords:** high responder, resistance training, mTOR, ribosome biogenesis, proteolysis

## Abstract

Limited evidence exists regarding differentially expressed biomarkers between previously-trained low versus high hypertrophic responders in response to resistance training. Herein, 30 college-aged males (training age 5 ± 3 years; mean ± SD) partook in 6 weeks of high-volume resistance training. Body composition, right leg vastus lateralis (VL) biopsies, and blood were obtained prior to training (PRE) and at the 3-week (W3) and 6-week time points (W6). The 10 lowest (LOW) and 10 highest (HIGH) hypertrophic responders were clustered based upon a composite hypertrophy score of PRE-to-W6 changes in right leg VL mean muscle fiber cross-sectional area (fCSA), VL thickness assessed via ultrasound, upper right leg lean soft tissue mass assessed via dual x-ray absorptiometry (DXA), and mid-thigh circumference. Two-way ANOVAs were used to compare biomarker differences between the LOW and HIGH clusters over time, and stepwise linear regression was performed to elucidate biomarkers that explained significant variation in the composite hypertrophy score from PRE to W3, W3 to W6, and PRE to W6 in all 30 participants. PRE-to-W6 HIGH and LOW responders exhibited a composite hypertrophy change of +10.7 ± 3.2 and -2.1 ± 1.6%, respectively (*p* < 0.001). Compared to HIGH responders, LOW responders exhibited greater PRE type II fCSA (+18%, *p* = 0.022). Time effects (*p* < 0.05) existed for total RNA/mg muscle (W6 > W3 > PRE), phospho (p)-4EBP1 (PRE > W3&W6), pan-mTOR (PRE > W3 < W6), p-mTOR (PRE > W3 < W6), pan-AMPKα (PRE > W3 < W6), pan-p70s6k (PRE > W3), muscle ubiquitin-labeled proteins (PRE > W6), mechano growth factor mRNA (W6 > W3&PRE), 45S rRNA (PRE > W6), and muscle citrate synthase activity (PRE > W3&W6). No interactions existed for the aforementioned biomarkers and/or other assayed targets (muscle 20S proteasome activity, serum total testosterone, muscle androgen receptor protein levels, muscle glycogen, or serum creatine kinase). Regression analysis indicated PRE type II fiber percentage (*R*^2^ = 0.152, β = 0.390, *p* = 0.033) and PRE type II fCSA (*R*^2^ = 0.207, β = -0.455, *p* = 0.019) best predicted the PRE-to-W6 change in the composite hypertrophy score. While our sample size is limited, these data suggest: (a) HIGH responders may exhibit more growth potential given that they possessed lower PRE type II fCSA values and (b) possessing a greater type II fiber percentage as a trained individual may be advantageous for hypertrophy in response to resistance training.

## Introduction

Low and high hypertrophic responders exist following weeks to months of structured resistance training. This interest was initially spurred by Bamman’s laboratory who reported that different hypertrophic response clusters existed following 16 weeks of resistance training (Bamman et al., 2007). Bamman’s laboratory (Kim et al., 2007; Petrella et al., 2008; Thalacker-Mercer et al., 2013; Stec et al., 2016), our laboratory (Mobley et al., 2018; Roberts et al., 2018b), and others (Davidsen et al., 2011; Ogasawara et al., 2016; Morton et al., 2018) have subsequently examined low and high responders following weeks to months of resistance training with the intent of deciphering biomarkers that exist between each cluster. Determining biomarkers or mechanisms which may explain the variance in the hypertrophic response to resistance training is important for a variety of reasons. First, follow-up studies can be performed in low responders in order to determine if increasing Caloric intake or various nutritional supplements (e.g., creatine or protein) improves the training response. Additionally, if low responders demonstrate an enhanced inflammatory or muscle damage profile to training, then reducing training stress or providing adjuvant therapies (e.g., anti-inflammatory strategies) may be a viable strategy to optimize training adaptations.

Various research groups have suggested that ribosome biogenesis is greater in high versus low responders following weeks to months of structured resistance training (Figueiredo et al., 2015; Stec et al., 2016; Mobley et al., 2018). There is also evidence to suggest that a greater degree of satellite cell proliferation and subsequent myonuclear accretion occurs in high versus low responders during training (Petrella et al., 2008), although contrary evidence exists when only examining college-aged males (Mobley et al., 2018). High responders may also possess an altered microRNA profile in skeletal muscle which acts to enhance insulin-like growth factor-1 (IGF-1) mRNA expression (Davidsen et al., 2011). Recent evidence also suggests that, in previously-trained college-aged men, skeletal muscle androgen receptor content is greater in high responders (Morton et al., 2018). Likewise, other studies have suggested that an up-regulation in muscle androgen receptor content is associated with skeletal muscle hypertrophy (Ahtiainen et al., 2011; Mitchell et al., 2013).

We recently published a study which subjected previously-trained college-aged males to 6 weeks of a very high-volume resistance training protocol (Haun et al., 2018b). Herein, we re-purposed said dataset to identify biomarkers related to myonuclear accretion, ribosome biogenesis, mTORc1 signaling, proteolysis, muscle damage, androgen signaling, and muscle metabolism which may have delineated the hypertrophic response to the 6-week training protocol. Unlike past reports which have examined biomarker differences between low and high hypertrophic responders that were previously untrained (Bamman et al., 2007; Davidsen et al., 2011; Ogasawara et al., 2016; Stec et al., 2016; Mobley et al., 2018), the current study is only one of two studies to make these observations in previously trained subjects (Morton et al., 2018). We hypothesized that HIGH responders would experience greater increases in myonuclear accretion as well as biomarkers related to ribosome biogenesis, androgen signaling, mTORc1 signaling, and mitochondrial biogenesis relative to LOW responders.

## Materials and Methods

### Ethics Statement and Study Design

Prior to engaging in data collection, this study was approved by the Institutional Review Board at Auburn University (Protocol #17-425 MR 1710). All subjects provided verbal and written consent, and this study conformed to the standards set by the latest revision of the Declaration of Helsinki. The testing procedures, resistance training protocol, molecular analyses, and histology methods employed herein are outlined below. Readers are referred to Haun et al. (2018b) for more in-depth descriptions of supplementation and nutritional recommendations.

### PRE, W3, and W6 Testing Sessions

During the prior to training (PRE), W3, and W6 testing sessions participants were instructed to arrive for testing batteries in an overnight fasted condition. At W3 and W6, testing sessions occurred 24 h following Friday workouts. Testing procedures pertinent to this dataset are described below, although other assessments were performed and described in greater detail elsewhere (Haun et al., 2018b). It should be noted that urine specific gravity assessments occurred first followed by body composition measures (DXA and ultrasound described below), and blood draws and muscle biopsies occurred last. Additionally, lower body strength testing occurred approximately 1 week prior to the PRE testing session procedures described below.

#### DXA and Ultrasound Assessments

At the beginning of each testing session participants submitted a urine sample (∼5 mL) to assess normal hydration specific gravity levels (1.005–1.020 ppm) using a handheld refractometer (ATAGO; Bellevue, WA, United States). Participants with a urine specific gravity ≥1.020 were asked to consume 400 ml tap water and were re-tested ∼20 min thereafter. Following hydration testing, height and body mass were assessed using a digital column scale (Seca 769; Hanover, MD, United States) with body masses and heights collected to the nearest 0.1 kg and 0.5 cm, respectively. Participants were then subjected to a full-body DXA scan (Lunar Prodigy; General Electric, Fairfield, CT, United States) while wearing general sports attire (i.e., athletic shorts or compression shorts and an athletic shirt). According to previous data published by our laboratory, the same-day reliability of the DXA during a test-calibrate-retest on 10 participants produced an intra-class correlation coefficient (ICC) of 0.998 for total body lean soft tissue mass (Kephart et al., 2016). After all DXA scans were complete, a technician (K.C.Y.) used a region of interest tool in the software to segment the upper right leg using standardized landmarks (enCORE version 15.00), and bone-free lean tissue mass (referred to as upper right leg lean soft tissue mass throughout) was generated through the Lunar software. Following DXA scans, participants were subjected to an ultrasound assessment to determine right leg mid-thigh muscle thickness with a 3 to 12 MHz multi-frequency linear phase array transducer (Logiq S7 R2 Expert; General Electric). Measurements were taken from the midway point between the iliac crest and patella of the right femur whereby participants were in a standing position and weight was placed on the left leg. Reliability for muscle thickness during a test–retest at PRE on 33 participants produced an ICC of 0.994 (Haun et al., 2018b). Notably, all DXA scans and ultrasound assessments were completed by the same investigators (M.A.R., and P.W.M., respectively) as suggested by previous research interventions (Lohman et al., 2009; Lockwood et al., 2017) in order to minimize variability in testing procedures.

#### Muscle Tissue and Blood Collection

Muscle biopsies were collected using a 5-gauge needle under local anesthesia as previously described (Haun et al., 2018b). Immediately following tissue procurement, ∼20–40 mg of tissue was embedded in cryomolds containing optimal cutting temperature (OCT) media (Tissue-Tek^®^, Sakura Finetek, Inc., Torrance, CA, United States). Embedding was performed whereby tissue was placed in cryomolds for cross-sectional slicing in a non-stretched state prior to rapid freezing. A desktop light microscope was used with a 4x objective to ensure tissue was situated appropriately within OCT cryomolds prior to freezing, and fine needles were used to make adjustments if needed. Cryomolds were then frozen using liquid nitrogen-cooled isopentane and subsequently stored at -80°C until histological analyses occurred. The remaining tissue was teased of blood and connective tissue, wrapped in pre-labeled foils, flash frozen in liquid nitrogen, and subsequently stored at -80°C until molecular analyses occurred. Venous blood samples were also collected into a 5 mL serum separator tube (BD Vacutainer, Franklin Lakes, NJ, United States) during the waiting period for local anesthesia to take effect. Creatine kinase (CK) activity, cortisol, and total testosterone were elected as serum targets of interest and more detailed methods of these assays are described below.

### Resistance Training Protocol

Participants were familiarized with the design of training and technical parameters during testing of 3RMs which occurred 3–7 days prior to PRE testing and training initiation. Strict technical parameters were employed for testing to ensure accurate reflections of strength under direct supervision of research staff holding the Certified Strength and Conditioning Specialist Certification from the National Strength and Conditioning Association.

Following 3RM testing and the PRE testing battery, resistance training occurred 3 days per week. Loads corresponding to 60% 1RM, based on three repetition maximum (3RM) testing, were programmed for each set of each exercise. Sets of 10 repetitions were programmed for each set of each exercise throughout the study. Exercises were completed one set at a time, in the following order during each training session: days 1 and 3 each week – barbell (BB) back squat, BB bench press, BB stiff-legged deadlift (SLDL), and an underhand grip cable machine pulldown exercise designed to target the elbow flexors and latissimus dorsi muscles (lat pulldown); day 2 of each week – BB back squat, BB overhead press, BB SLDL, and lat pulldown. The 3 day per week protocol involved a progressive increase from 10 sets per week to 32 sets per week for each exercise. Thus, on the last week of training participants performed 32 sets of 10 repetitions of BB back squats, 32 sets of 10 repetitions of BB bench press and OH press combined, 32 sets of 10 repetitions of BB SLDL, and 32 sets of 10 repetitions of lat pulldowns. Readers are referred to Haun et al. (2018b) for more in-depth descriptions of training.

### Muscle Tissue Processing

For protein and RNA analyses tissue foils were removed from -80°C and crushed using a liquid nitrogen-cooled mortar and pestle. For protein analysis, ∼30 mg of powdered tissue was placed in 1.7 mL microcentrifuge tubes containing 500 μL of ice-cold cell lysis buffer [20 mM Tris-HCl (pH 7.5), 150 mM NaCl, 1 mM Na_2_EDTA, 1 mM EGTA, 1% Triton; Cell Signaling, Danvers, MA, United States] pre-stocked with protease and Tyr/Ser/Thr phosphatase inhibitors (2.5 mM sodium pyrophosphate, 1 mM β-glycerophosphate, 1 mM Na_3_VO_4_, 1 μg/mL leupeptin). Samples were then homogenized by hand using tight micropestles, insoluble proteins were removed with centrifugation at 500 *g* for 5 min, and obtained sample lysates were stored at -80°C prior to Western blotting and other biochemical assays (described below).

For total RNA analysis, ∼15–30 mg of powdered tissue was weighed using an analytical scale with a sensitivity of 0.001 g (Mettler-Toledo; Columbus, OH, United States). Tissue was then homogenized in 1.7 mL microcentrifuge tubes containing 500 μL of Ribozol (Ameresco; Solon, OH, United States) via micropestle manipulation and RNA isolation was performed per manufacturer recommendations. Total RNA concentrations were then determined in duplicate using a NanoDrop Lite spectrophotometer (Thermo Fisher Scientific; Waltham, MA, United States), and total RNA per unit muscle weight was used as a surrogate for ribosome density as in past publications (Nader et al., 2005; Mobley et al., 2016).

### Immunohistochemistry for fCSA and Myonuclear Number Determination

Methods for immunohistochemistry have been employed previously in our laboratory and described elsewhere (Hyatt et al., 2015; Martin et al., 2016; Mobley et al., 2017a). Briefly, sections from OCT-preserved samples were cut at a thickness of 8 μm using a cryotome (Leica Biosystems; Buffalo Grove, IL, United States) and were adhered to positively-charged histology slides. Once all samples were sectioned, batch processing occurred for immunohistochemistry. During batch processing sections were air-dried at room temperature for up to 10 min, permeabilized in a phosphate-buffered saline (PBS) solution containing 0.5% Triton X-100, and blocked with 100% Pierce Super Blocker (Thermo Fisher Scientific) for 25 min. Sections were then incubated for 20 min with a pre-diluted commercially-available rabbit anti-dystrophin IgG antibody solution (catalog #: GTX15277; Genetex, Inc., Irvine, CA, United States) and spiked in mouse anti-myosin I IgG (catalog #: A4.951 supernatant; Hybridoma Bank, Iowa City, IA, United States; 40 μL added per 1 mL of dystrophin antibody solution). Sections were then washed for 2 min in PBS and incubated in the dark for 20 min with a secondary antibody solution containing Texas Red-conjugated anti-rabbit IgG (catalog #: TI-1000; Vector Laboratories, Burlingame, CA, United States), and Alexa Fluor 488-conjugated anti-mouse IgG (catalog #: A-11001; Thermo Fisher Scientific) (∼6.6 μL of all secondary antibodies per 1 mL of blocking solution). Sections were washed for 5 min in PBS, air-dried, and mounted with fluorescent media containing 4,6-diamidino-2-phenylindole (DAPI; catalog #: GTX16206; Genetex, Inc.). Following mounting, slides were stored in the dark at 4°C until digital immunofluorescent images were obtained.

After staining was performed on all sections, digital images were captured using a fluorescent microscope (Nikon Instruments, Melville, NY, United States) at a 10x objective. Approximate exposure times were 600 ms for TRITC and FITC imaging and 80 ms for DAPI imaging. This staining method allowed the identification of cell membranes (detected by the Texas Red filter), type I fiber green cell bodies (detected by the FITC filter), type II fiber black cell bodies (unlabeled), and myonuclei (detected by the DAPI filter). Standardized measurements of types I and II fiber cross-sectional areas (fCSAs) were performed using the open-sourced software CellProfiler^TM^ (Carpenter et al., 2006) per modified methods previously described whereby the number of pixels counted within the border of each muscle fiber were converted to a total area (μm^2^) (Mobley et al., 2017a). A calibrator slide containing a 250,000 μm^2^ square image was also captured, and pixels per fiber from imaged sections were converted to area using this calibrator image. On average, 113 ± 26 fibers per cross-section were identified for analysis at each sampling time. A *post hoc* experiment performed in our laboratory to examine potential differences in fCSA measurements between sections on the same slide (*n* = 27 slides) revealed strong reliability using this method (ICC = 0.929). Measurements of fiber type-specific myonuclear number were also performed using a custom script in CellProfiler^TM^ which discriminates the fiber border that corresponded to each myonucleus (Mobley et al., 2017a).

### Western Blotting

Whole-tissue sample lysates obtained through cell lysis buffer processing (described above) were batch process-assayed for total protein content using a BCA Protein Assay Kit (Thermo Fisher Scientific). Lysates were then prepared for Western blotting using 4x Laemmli buffer at 1 μg/μL. Following sample preparation, 18 μL samples were loaded onto 4–15% SDS-polyacrylamide gels (Bio-Rad; Hercules, CA, United States) and subjected to electrophoresis (180 V for 45–60 min) using pre-made 1x SDS-PAGE running buffer (Ameresco; Framingham, MA, United States). Proteins were then transferred (200 mA for 2 h) to polyvinylidene difluoride membranes (Bio-Rad), Ponceau stained and imaged to ensure equal protein loading between lanes. Membranes were then blocked for 1 h at room temperature with 5% non-fat milk powder in Tris-buffered saline with 0.1% Tween-20 (TBST; Ameresco). Rabbit anti-human phospho-p70s6k (Thr389) (1:1,000; catalog #: 9234; Cell Signaling), rabbit anti-human pan p70s6k (1:1,000; catalog #: 2708; Cell Signaling), rabbit anti-human phospho-4EBP1 (Thr37/46) (1:1,000; catalog #: 2855; Cell Signaling), rabbit anti-human pan 4EBP1 (1:1,000; catalog #: 9644; Cell Signaling), rabbit anti-human phospho-mTOR (Ser2448) (1:1,000; catalog #: 2971; Cell Signaling), rabbit anti-human pan mTOR (1:1,000; catalog #: 2972; Cell Signaling), rabbit anti-human phospho-AMPKα (Thr172) (1:1,000; catalog #: 2535; Cell Signaling), rabbit anti-human pan AMPKα (1:1,000; catalog #: 2532; Cell Signaling), rabbit anti-human androgen receptor (1:1,000; catalog #: 5153; Cell Signaling) and rabbit anti-human ubiquitin (1:1,000; catalog #: 3933; Cell Signaling) were incubated with membranes overnight at 4°C in TBST with 5% bovine serum albumin (BSA). The following day, membranes were incubated with horseradish peroxidase-conjugated anti-rabbit (catalog #: 7074; Cell Signaling) in TBST with 5% BSA at room temperature for 1 h (1:2,000). Membrane development was performed using an enhanced chemiluminescent reagent (Luminata Forte HRP substrate; EMD Millipore, Billerica, MA, United States), and band densitometry was performed using a gel documentation system and associated densitometry software (UVP, Upland, CA, United States). Densitometry values for all targets were divided by whole-lane Ponceau densities. All Western blotting data are expressed as relative expression units (REUs).

### Real-Time PCR

Two μg of RNA was reverse transcribed into cDNA for RT-PCR analysis with cDNA synthesis reagents (Quanta Biosciences, Gaithersburg, MD, United States) per the manufacturer’s recommendations. RT-PCR was performed using gene-specific primers and SYBR green chemistry (Quanta Biosciences). Primer sequences used were as follows: 45S pre-rRNA forward primer 5′-GAACGGTGGTGTGTCGTT-3′, reverse primer 5′-GCGTCTCGTCTCGTCTCACT-3′; mechano growth factor (MGF): forward primer 5′-CGAAGTCTCAGAGAAGGAAAGG-3′, reverse primer 5′-ACA GGTAACTCGTGCAGAGC-3′; myostatin (MSTN): forward primer 5′-GACCAGGAGAAGATGGGCTGAATCCGTT-3′, reverse primer 5′-CTCATCACAGTCAAGACCAAAATCCCTT-3′; beta-2-microglobulin (B2M, housekeeping gene 1): forward primer 5′-ATGAGTATGCCTGCCGTGTGA-3′ reverse primer 5′-GGCATCTTCAAACCTCCATG-3′; cyclophilin (PPIA, housekeeping gene 2): forward primer 5′-CGATGTCTCAGAGCACGAAA-3′, reverse primer 5′-CCCACCTGTTTCTTCGACAT-3′. PCR calculations were performed as previously described by our laboratory (Haun et al., 2018a). Briefly, 2^-ΔCq^ values for each gene of interest at each time point were calculated whereby ΔCq = gene of interest Cq – geometric mean housekeeping gene Cq values. All W3 and W6 values for a given mRNA target were then normalized to PRE values within subject, and PCR data were expressed as fold-change scores. Prior melt curve analyses from our laboratory confirmed that only one RT-PCR product was obtained with the primer sets being used.

### Citrate Synthase Activity Assay

Whole-tissue sample lysates obtained through cell lysis buffer processing (described above) were batch processed for citrate synthase activity as previously described (Roberts et al., 2018b), and this metric was used as a surrogate for mitochondrial content per the findings of Larsen et al. (2012) suggesting citrate synthase activity exhibits a strong correlation with electron micrograph images of mitochondrial content (*r* = 0.84, *p* < 0.001). The assay utilized is based on the reduction of 5,50-dithiobis (2- nitrobenzoic acid) (DTNB) at 412 nm (extinction coefficient: 13.6 mmol/L/cm) coupled to the reduction of acetyl-CoA by the citrate synthase reaction in the presence of oxaloacetate. Briefly, 12.5 μg of skeletal muscle protein were added to a mixture composed of 0.125 mol/L Tris-HCl (pH 8.0), 0.03 mmol/L acetyl-CoA, and 0.1 mmol/L DTNB. The reaction was initiated by the addition of 5 μL of 50 mmol/L oxaloacetate and the absorbance change was recorded for 1 min. The average coefficient of variation for all duplicates was ∼8%.

### 20S Proteasome Activity Assay

Forty μg of skeletal muscle protein from whole-tissue sample lysates obtained through cell lysis buffer processing (described above) were batch processed for 20S proteasome activity as previously described using commercially available fluorometric kits (catalog #: APT280; Millipore Sigma; Burlington, MA, United States) per the manufacturer’s instructions which are similar to methods previously published by our laboratory (Mobley et al., 2017b). Assay readings are presented as relative fluorometric units (RFUs). The average coefficient of variation for all duplicates was 8.7%.

### Glycogen Assay

Whole-tissue sample lysates were batch processed for glycogen determination using commercially-available fluorometric kits (catalog #: MAK016; Millipore Sigma) per the manufacturer’s instructions. This assay was piloted with whole tissue lysates, frozen wet tissue, and lyophilized tissue, and all three methods yielded similar results. Thus, given that whole tissue lysates were available for most participants, we opted to assay this tissue fraction. A standard curve was used to determine glycogen content of whole-tissue sample lysates, this value was multiplied by the starting cell lysis buffer volume (500 μL) to derive total glycogen content per sample, and the resultant value was divided by input wet muscle weights to obtain nmol glycogen/mg wet muscle weight. The average coefficient of variation for all duplicates was 9.8%.

### Serum Assays

Upon blood collection, serum tubes were centrifuged at 3,500 *g* for 5 min at room temperature. Aliquots were then placed in 1.7 mL polypropylene tubes and stored at -80°C until batch-processing. An activity assay was used to determine serum levels of CK (Bioo Scientific; Austin, TX, United States). In cases where samples were missing or where the standard curve indicated that blood levels were negative, values were not considered in the analysis. Commercially-available ELISA kits (ALPCO Diagnostics; Salem, NH, United States) were used to assay serum total testosterone and cortisol. All kits were performed according to manufacturer’s instructions and plates were read using a 96-well spectrophotometer (BioTek, Winooski, VT, United States). The average coefficient of variation of duplicate values for each target were as follows: serum testosterone = 6.9%, serum cortisol = 2.8%, and serum CK activity = 1.4%.

### Statistical Analysis and Responder Clustering

Statistical tests were performed in RStudio (Version 1.0.143) and SPSS (Version 25). Regarding HIGH and LOW hypertrophic cluster analysis we adopted methods similar to Davidsen et al. (2011), as well a recent publication from our laboratory in untrained participants (Roberts et al., 2018b), wherein the 10 lowest (LOW) and 10 highest (HIGH) hypertrophic responders were clustered based upon a composite hypertrophy score of PRE-to-W6 percent changes in:

(a) right leg VL muscle thickness assessed via ultrasound
(b) upper right leg lean soft tissue assessed via DXA
(c) right leg mid-thigh circumference
(d) right leg VL mean (type I and type II) muscle fCSA.

**FIGURE 1:**
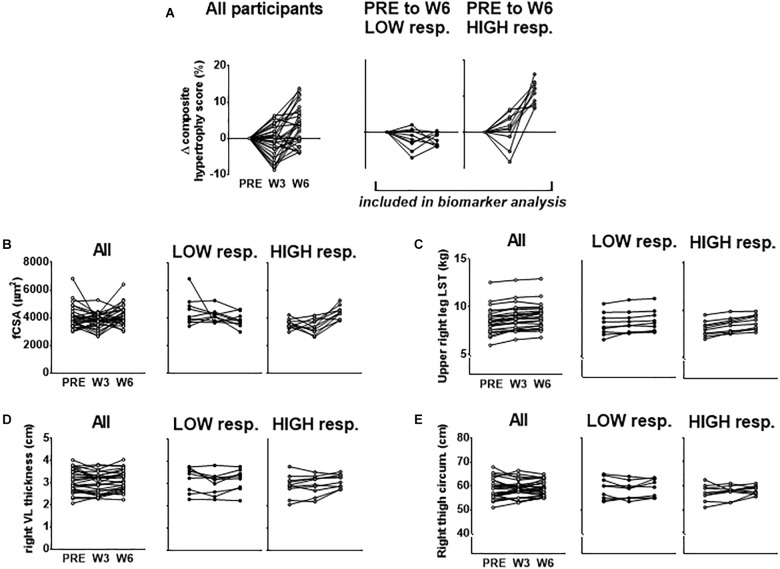
PRE to W6 percent changes in hypertrophic indices used to cluster LOW and HIGH responders. **(A)** PRE to W3 and PRE to W6 changes in the composite hypertrophy score in all participants as well as LOW (*n* = 10) and HIGH (*n* = 10) responders. Response clusters were ranked according to the PRE to W6 composite score which was made up of percent changes in mean fiber cross sectional area (fCSA), upper right leg DXA lean soft tissue (LST), right leg vastus lateralis (VL) thickness, and right thigh circumference. Raw values for each of these composite score variables for all subjects as well as response clusters are presented in **(B–E)**.

Our rationale for using multiple indices to delineate LOW and HIGH responders is due to previous data from our laboratory demonstrating that defining clusters based upon VL thickness alone did not yield between-cluster differences in 3RM back squat strength changes following 12 weeks of resistance training (Mobley et al., 2018). However, using a combination of DXA-based, histology-based, and ultrasound-based hypertrophic indices to cluster LOW and HIGH responders in this same subject pool yielded between-cluster differences in this strength metric [HIGH pre-to-post delta (Δ)3RM squat = 42 ± 3 kg, LOW Δ3RM squat = 31 ± 9 kg, respectively; *p* = 0.005] (Roberts et al., 2018b). Moreover, we sought to cluster in an unbiased manner toward any hypertrophic assessment and felt equally weighting each variable in forming the composite score by using percent change was appropriate. Further, since many biomarkers were derived from the biopsy sample of the VL, and fCSA typically produces greater percent changes than other levels of hypertrophic assessment, allowing fCSA percent changes to leverage the composite score served as additional rationale. Figure 1 below demonstrates the percent change score for the composite hypertrophy variable as well as absolute values for a–d above between clusters.

All dependent variable comparisons over time were analyzed between LOW and HIGH clusters using 2 × 3 (cluster [LOW, HIGH] × time [PRE, W3, W6]) mixed factorial repeated measures ANOVAs with the exception of histology data and self-reported food log data which were analyzed using 2 × 2 (cluster [LOW, HIGH] × time [PRE, W6]) models. If a significant cluster × time interaction was observed, LSD *post hoc* tests were performed within each cluster and between clusters at each time point. Significance wasestablished at *p* < 0.05.

Aside from comparing the 10 HIGH and 10 LOW responders, stepwise linear regression was also performed to predict the PRE to week 3 (W3), W3 to W6, and PRE to W6 composite hypertrophy score responses in all 30 participants using PRE biomarker data as well as percent changes in biomarkers from PRE to W3, W3 to W6 and PRE to W6. For regression analysis, the following variables at PRE were measured and considered for inclusion in the overall analysis as predictors: (a) PRE type I fiber percentage, (b) PRE type II fiber percentage, (c) PRE type I fCSA, (d) PRE type II fCSA, (e) PRE type I fiber myonuclear number, (f) PRE type II fiber myonuclear number, (g) PRE muscle 20S proteasome activity, (h) PRE muscle glycogen, (i) PRE citrate synthase activity, (j) PRE serum creatine kinase activity, (k) PRE serum testosterone, (l) PRE serum cortisol, (m) PRE muscle pan 4EBP1, (n) PRE muscle p-4EBP1, (o) PRE muscle pan mTOR, (p) PRE muscle p-mTOR, (q) PRE muscle pan AMPK, (r) PRE muscle p-AMPK, (s) PRE muscle pan p70s6k, (t) PRE muscle p-p70s6k, (u) PRE muscle pan poly Ub, (v) PRE muscle RNA, (w) PRE muscle MGF mRNA, (x) PRE muscle 45S rRNA, and (y) PRE muscle MSTN mRNA. Percent change scores were also calculated for the predictors above, except for fiber percentages and fCSA to avoid multicollinearity, and were considered for inclusion in models explaining variation in the hypertrophic response from PRE to W3 (PRE-W3), W3 to W6 (W3–W6), and PRE to W6 (PRE-W6). Percent change variables are named below as time point to time point percent change (e.g., PRE-W3 muscle p-mTOR percent change). Critically, although baseline predictors were based on raw values, percent change values were calculated for all biomarker predictors for consistent, standardized inference (e.g., percent change in x relative to percent change in y). In order to construct more meaningful models and due to statistical power, bivariate correlations were completed prior to stepwise regression for each predictor and the dependent variable of the model to eliminate predictors weakly correlated with the dependent variable at each level of time (*r* < 0.3). Upon identification of predictors correlating with the dependent variable beyond *r* = 0.3 and satisfaction of assumptions tests, analysis proceeded. Measurement of each predictor variable occurred for each subject at each time point (PRE, W3, and W6), except for AR protein content where only HIGH and LOW clusters were examined as a *post hoc* analysis (further described below) or in the case of a lack of sample. Power analyses in RStudio using general linear model parameters in the “pwr” package (Version 1.2–1) revealed ≥ 80% power (power = 1 - β) for the discovery of large effects when models included 30 subjects and up to 3 predictor variables. However, analyses were underpowered to identify small and medium effects (<80%). Therefore, we intended to construct parsimonious models with fewer predictors to identify large effects and avoid commission of type 1 or 2 errors. Additionally, effort was made to include all datum in the analysis that were biologically plausible and outlier detection was only considered if datum exceeded 3 standard deviations from the mean of each individual predictor. However, if datum exceeded 3 standard deviations from the mean but were considered biologically possible, analysis proceeded. To assist in the identification of potential outliers and to further protect against erroneous conclusions, assumptions tests and regression diagnostics were also performed. These included: (a) Shapiro–Wilk tests for normality of residual distributions, (b) Levene’s tests of homogeneity of variance between levels of time for each predictor, (c) homogeneity of regression slopes, and (d) multicollinearity assessments. If Levene’s test was violated, a Greenhouse–Geisser adjustment to degrees of freedom was made for more conservative *p*-value inference. Homogeneity of regression slopes were evaluated by examining predictor variable’s individual relationship with the dependent variable in order to build more meaningful models. This assessment was to ensure predictors in models had consistently positive or negative relationships with the composite hypertrophy score which was verified through bivariate correlations. Multicollinearity was assessed through examining which predictors were correlated and models were also inspected via variance inflation factor scores (VIF < 10). If predictor variables were strongly correlated (*r* > 0.5) or VIF scores exceeded 10, predictors were eliminated, or separate models were constructed to examine the explained variation by individual predictors. For clarity, only the strongest predictors from the overall stepwise regression analysis are discussed in the results section below.

The sample size for the overall stepwise regression analysis was 30 subjects, while specific sample sizes for each analysis and cluster analysis are reported in figures or text where appropriate. All data for this study are provided in Supplementary File S1.

## Results

### Baseline Characteristics and Training Volume Differences Between Clusters

Pre-training cluster differences in age, self-reported resistance training age, body mass, body composition, fiber type, pre-training three repetition back squat strength, and back squat training volume differences throughout the intervention are presented in Table 1. Notably, there were no significant differences between clusters regarding these variables (*p* > 0.05).

**Table 1:** Baseline characteristics at PRE and back squat training volume between clusters.

Variable	LOW (*n* = 10)	HIGH (*n* = 10)	*p*-value
Age (years)	22 ± 1	21 ± 2	0.376
Training age (years)	6 ± 2	6 ± 2	1.000
Body mass (kg)	83.1 ± 12.8	78.8 ± 8.0	0.381
DXA LST (kg)	65.1 ± 9.7	62.2 ± 5.9	0.430
DXA FM (kg)	14.5 ± 4.9	13.5 ± 4.9	0.649
Type II fiber (%)	50 ± 14	59 ± 17	0.189
3RM back squat (kg)	135 ± 14	127 ± 23	0.342
Total back squat training volume (kg) from weeks 1 to 6	111,821 ± 12,962	106,610 ± 18,679	0.478
**Number of participants in different nutritional groups from Haun et al. (2018a)**		
GWP	5	3	Chi-square *p*-value
MALTO	1	3	
WP	4	4	0.199


*These data demonstrate that pre-training age, training age, body composition, muscle fiber type, and three repetition maximum (3RM) back squat strength, and back squat training volume during the 6-week intervention did not differ between LOW and HIGH responders. Moreover, the LOW and HIGH responders were not influenced by different nutritional interventions reported in the study by Haun et al. (2018b). All data are presented as mean ± standard deviation values. DXA LST, lean soft tissue assessed using dual x-ray absorptiometry; DXA FM, fat mass assessed using dual x-ray absorptiometry. MALTO, participants that supplemented once daily with 30 g of maltodextrin; WP, participants that supplemented once daily with 25 g of whey protein concentrate; GWP, participants that supplemented 25 g/d WP during week 1, 50 g/d during week 2, 75 g/d during week 3, 100 g/d during week 4, 125 g/d during week 5, and 150 g/d during week 6.*

### Self-Reported Macronutrient Intakes Between Clusters

PRE and W6 energy and absolute as well as relative (per kg) macronutrient intake differences between clusters are presented in Table 2. There were no significant cluster effects, time effects, or cluster × time interactions for these variables.

**Table 2:** Self-reported macronutrient intakes between clusters at PRE and week 6.

Variable Group	PRE mean ± SD	W6 mean ± SD	Statistics
**Energy intake (kcal/d)**		
LOW	2,874 ± 499	2,459 ± 1,015	Cluster *p* = 0.454
HIGH	3,067 ± 186	2,699 ± 524	Time *p* = 0.096
			C × T *p* = 0.918
**Energy intake (kcal/kg/d)**		
LOW	34.1 ± 6.0	28.7 ± 11.2	Cluster *p* = 0.122
HIGH	39.2 ± 3.0	33.5 ± 6.2	Time *p* = 0.064
			C × T *p* = 0.963
**Protein intake (g/d)**		
LOW	177 ± 29	186 ± 100	Cluster *p* = 0.511
HIGH	194 ± 38	204 ± 41	Time *p* = 0.641
			C × T *p* = 0.972
**Protein intake (g/kg/d)**		
LOW	2.1 ± 0.2	2.2 ± 1.1	Cluster *p* = 0.187
HIGH	2.5 ± 0.8	2.6 ± 0.6	Time *p* = 0.824
			C × T *p* = 0.975
**Carbohydrate intake (g/d)**		
LOW	263 ± 95	223 ± 93	Cluster *p* = 0.347
			Time *p* = 0.230
HIGH	290 ± 30	259 ± 70	C × T *p* = 0.870
**Carbohydrate intake (g/kg/d)**		
LOW	3.2 ± 1.2	2.6 ± 1.0	Cluster *p* = 0.130
HIGH	3.7 ± 0.3	3.2 ± 0.8	Time *p* = 0.172
			C × T *p* = 0.913
**Fat intake (g/d)**		
LOW	126 ± 27	98 ± 39	Cluster *p* = 0.976
HIGH	124 ± 30	101 ± 25	Time *p* = 0.013 (W1 > W6)
			C × T *p* = 0.756
**Fat intake (g/kg/d)**		
LOW	1.5 ± 0.3	1.2 ± 0.5	Cluster *p* = 0.576
HIGH	1.3 ± 0.6	1.1 ± 0.5	Time *p* = 0.012 (W1 > W6)
			C × T *p* = 0.704


*These data demonstrate that PRE and week 6 (W6) self-reported energy and macronutrient intakes were similar between clusters. Notably, only *n* = 6 HIGH and *n* = 9 LOW were included in the analysis given that the other participants per cluster did not regularly self-report food intakes; refer to Haun et al. (2018a) for a more in-depth description of dietary analysis and subject removal due to non-compliant reporting. All data are presented as mean ± standard deviation values. Additionally, W6 data contains added supplemental macronutrients. C × T, cluster by time interaction.*

### Cluster Differences in Types I and II fCSA and Myonuclear Number

**FIGURE 2:**
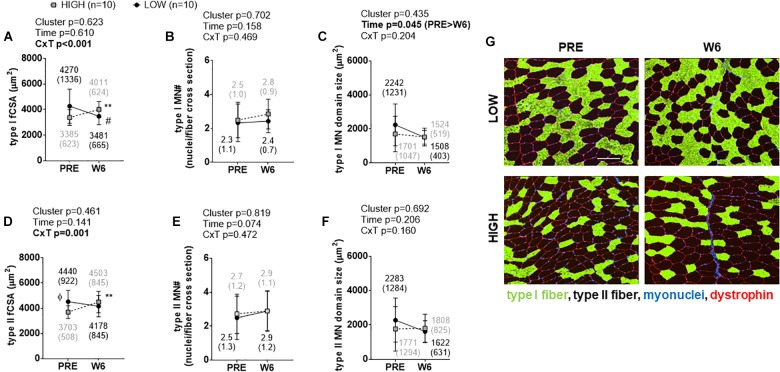
PRE to W6 changes in type I and type II fCSA and myonuclear number as well as myonuclear domain size between LOW and HIGH responders. **(A)** Type I muscle fCSA in LOW versus HIGH responders; values decreased in LOW responders from PRE to W6 (^#^*p* = 0.019) and increased in HIGH responders from PRE to W6 (^∗∗^*p* = 0.003). **(B)** No significant time effect, cluster effect, or cluster × time (C × T) interaction existed for type I fiber myonuclear number (MN#). **(C)** A significant time effect, but not cluster effect or C × T interaction existed for type I fiber myonuclear domain size. **(D)** Type II muscle fCSA was 18% greater at PRE in LOW versus HIGH responders (Φ, *p* = 0.022), but values only increased in HIGH responders from PRE to W6 (^∗∗^*p* = 0.005). **(E)** No main effects or interaction existed for PRE to W6 changes in type II fiber MN#. **(F)** No significant main effects or C × T interaction existed for type II fiber myonuclear domain size. Values presented in line graphs are mean (standard deviation) values. **(G)** Representative 10x objective images from a low and high responder (white scale bar = 200 μm).

Significant cluster × time interactions were observed for type I fCSA (*p* < 0.001; Figure 2A) and type II fCSA (*p* = 0.001; Figure 2D). No PRE differences existed between clusters for type I fCSA values, although type II fCSA values were greater at PRE and W3 in LOW versus HIGH responders (*p* < 0.05). Percent changes in types I and II fCSA from PRE to W6 were significantly different between clusters (*p* < 0.001). Type I fCSA decreased by -15.62 ± 13.66% and type II fCSA by -7.44 ± 11.47% in LOW responders whereas type I fCSA increased by 20.25 ± 18.27% and type II fCSA by 21.96 ± 20.17% in HIGH responders. No significant time effects, cluster effects, or cluster × time interactions existed for type I or type II fiber myonuclear number (Figure 2B,E). A significant time effect existed for type I myonuclear domain size (PRE > W6, *p* = 0.045; Figure 2C), but no cluster effect or interaction existed. No significant time effect, cluster effect, or interaction existed for type II fiber myonuclear domain size (Figure 2F). Representative images from a LOW and HIGH responder are presented in Figure 2G.

### Cluster Differences in Ribosome Biogenesis Markers

**FIGURE 3:**
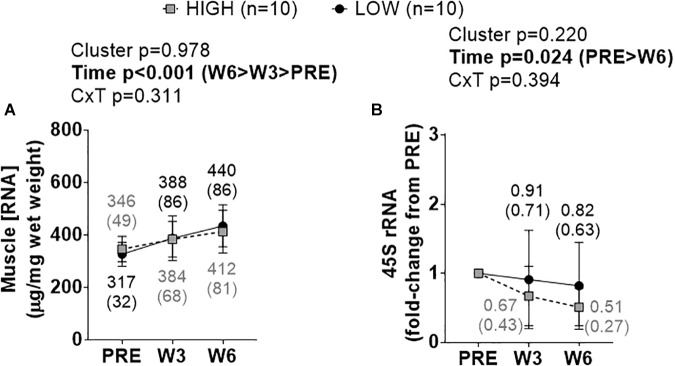
PRE, W3, and W6 ribosome biogenesis marker differences between LOW and HIGH responders. **(A)** A significant time effect existed for increases in total RNA density (i.e., ribosome density). **(B)** A significant time effect existed for decreases in 45S rRNA fold-change scores. Values presented in line graphs are mean (standard deviation) values.

A significant time effect existed for increases in total RNA density (i.e., ribosome density) (W3&6 > PRE, *p* < 0.001), but no significant cluster effect or cluster × time interaction existed (Figure 3A). A significant time effect also existed for decreases in 45S rRNA fold-change scores (W6 < PRE, *p* = 0.008), but no significant cluster effect or cluster × time interaction existed (Figure 3B).

### Cluster Differences in mTORc1 Signaling Markers

**FIGURE 4:**
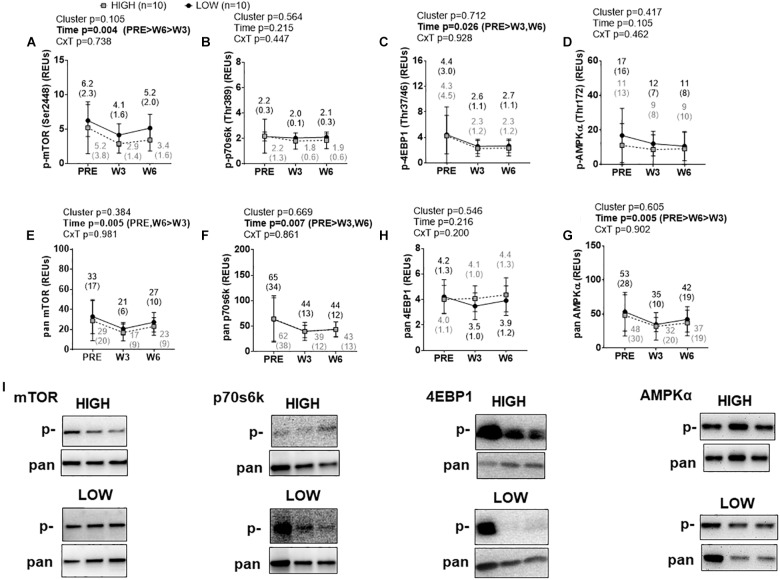
PRE, W3, and W6 mTORc1 signaling marker differences between LOW and HIGH responders. **(A–D)** Phosphorylated (p-) mTORc1 targets. There were no significant cluster effects or cluster × time interactions for said markers. p-mTOR and p-4EBP1 demonstrated significant time effects. **(E–H)** Total (pan) mTOR, p70s6k, 4EBP1, and AMPKα. Again, there were no significant cluster effects or cluster × time interactions for said markers, although there were significant time effects for pan mTOR, pan p70s6k, and pan AMPKα. Values presented in line graphs are mean (standard deviation) values. **(I)** Representative Western blot images for a low and high responder.

There were no significant cluster effects or cluster × time interactions for p-mTOR (Ser2448) (Figure 4A), p-p70s6k (Thr389) (Figure 4B), p-4EBP1 (Thr37/46) (Figure 4C), or p-AMPKα (Thr172) (Figure 4D). p-4EBP1 exhibited a significant time effect (PRE > W3&W6, *p* < 0.05 at each comparison) as did p-mTOR (PRE > W6 > W3, *p* < 0.05 at each comparison). There were no significant cluster effects or cluster × time interactions for pan mTOR (Figure 4E), pan p70s6k (Figure 4F), pan 4EBP1 (Figure 4G), or pan AMPKα (Figure 4H). However, there were significant time effects whereby pan mTOR exhibited a decrease at W3 relative to PRE and an increase from W3 to W6 (*p* < 0.05 at each comparison), pan p70s6k exhibited a decrease at W3 and W6 relative to PRE (*p* < 0.05 at each comparison), and pan AMPKα exhibited a decrease at W3 relative to PRE and an increase from W3 to W6 (*p* < 0.05 at each comparison). Representative Western blots for these targets are presented in Figure 4I.

### Cluster Differences in Biomarkers Related to Muscle Damage and Proteolysis

**FIGURE 5:**
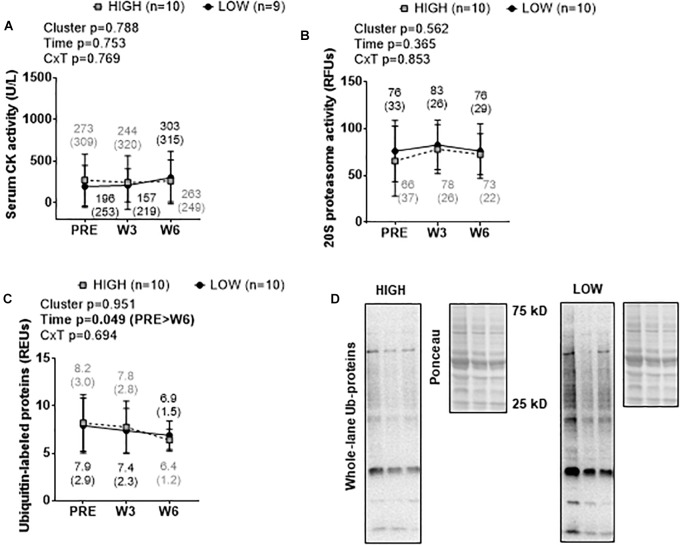
PRE, W3, and W6 muscle damage and proteolysis marker differences between LOW and HIGH responders. No significant cluster effects or cluster × time interactions existed for serum CK activity **(A)**, muscle 20S proteasome activity **(B)**, or muscle ubiquitin-labeled protein levels **(C)**. A significant time effect existed for muscle ubiquitin-labeled protein levels. Values presented in line graphs are mean (standard deviation) values. **(D)** Representative Western blot image for a low and high responder.

There were no significant cluster effects or cluster × time interactions for serum CK activity (Figure 5A), muscle 20S proteasome activity (Figure 5B), or muscle ubiquitin-labeled protein levels (Figure 5C). A significant time effect was observed for muscle ubiquitin-labeled protein levels where levels were lower at W6 than PRE (*p* = 0.044). Representative Western blots for ubiquitin-labeled proteins are presented in Figure 5D.

### Cluster Differences in MGF and MSTN mRNA Levels

**FIGURE 6:**
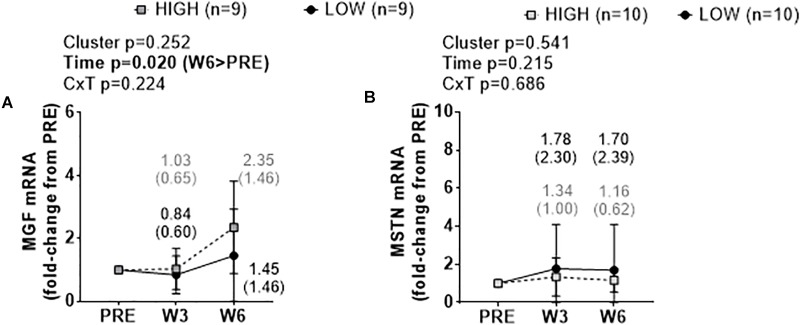
PRE, W3, and W6 growth factor mRNA expression differences between LOW and HIGH responders. No significant cluster effects or cluster × time interactions existed for fold-change scores in muscle mechano growth factor (MGF) mRNA **(A)** or myostatin (MSTN) mRNA **(B)**. There was a significant time effect for MGF mRNA. Values presented in line graphs are mean (standard deviation) values.

There were no significant cluster effects or cluster × time interactions for fold-change scores in muscle MGF mRNA (Figure 6A) or MSTN mRNA (Figure 6B). There was a significant time effect for MGF mRNA whereby W6 values were greater than PRE (*p* = 0.020).

### Cluster Differences in Androgen Signaling Markers

**FIGURE 7:**
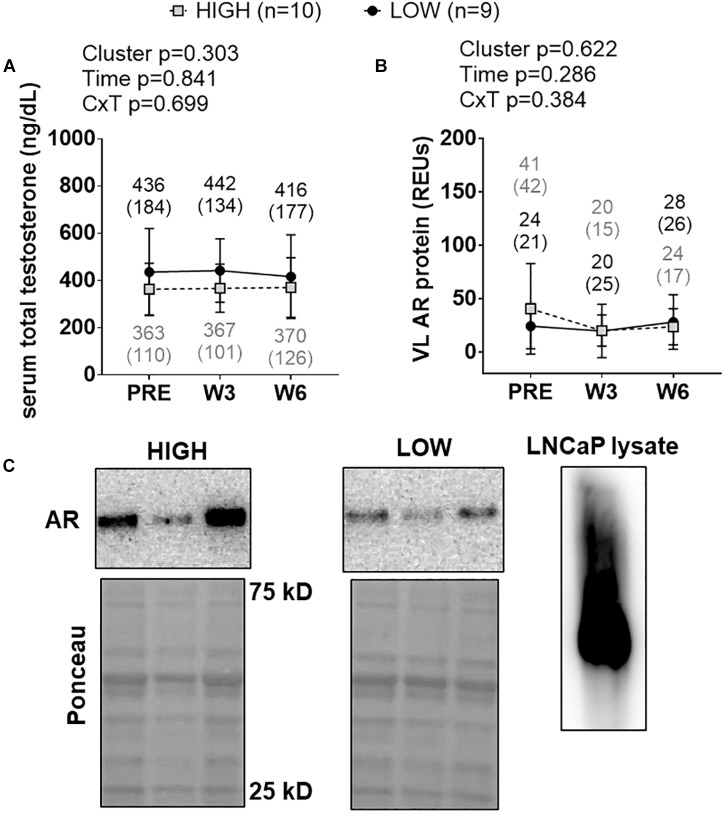
PRE, W3, and W6 androgen signaling biomarker differences between LOW and HIGH responders. There were no significant cluster effects, time effects or cluster × time interactions for serum total testosterone levels **(A)** or muscle androgen receptor (AR) protein levels **(B)**. Cluster means at each time point are presented above line graphs as mean (standard deviation) values. Values presented in line graphs are mean (standard deviation) values. **(C)** Representative Western blot image for a low and high responder. Right inset of **(C)** demonstrates that our AR antibody yielded one prominent band for LNCaP cell lysates, and this band was in line with muscle AR content that was quantified.

There were no significant cluster effects, time effects or cluster × time interactions for serum total testosterone levels (Figure 7A) or muscle AR protein levels (Figure 7B). Representative Western blots for AR are presented in Figure 7C.

### Cluster Differences in Skeletal Muscle Glycogen and Citrate Synthase Activity Levels

**FIGURE 8:**
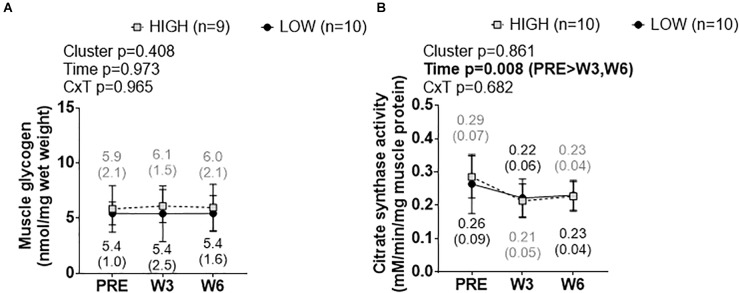
PRE, W3, and W6 muscle glycogen and citrate synthase activity level differences between LOW and HIGH responders. **(A)** No significant cluster effect, time effect or cluster × time interaction existed for muscle glycogen levels. **(B)** No significant cluster effect or cluster × time interaction existed for muscle citrate synthase activity levels, although there was a significant time effect. Values presented in line graphs are mean (standard deviation) values.

There was no significant cluster effect, time effect, or cluster × time interaction for muscle glycogen levels (Figure 8A). Likewise, there was no significant cluster effect or cluster × time interaction for muscle citrate synthase activity levels (Figure 8B), although there was a significant time effect whereby W3 values and W6 values were lower than PRE (*p* < 0.05 at each comparison).

### Stepwise Linear Regression to Establish Predictors of Hypertrophy

**FIGURE 9:**
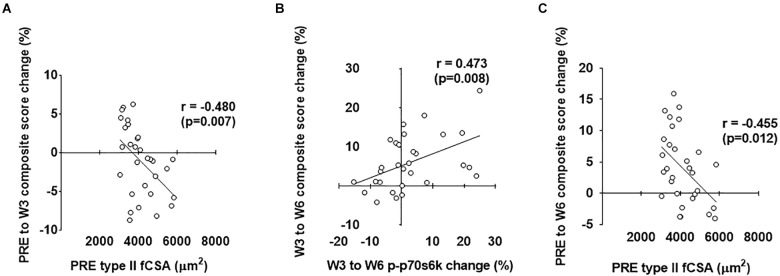
Strongest predictors of PRE to W3, W3 to W6, and PRE to W6 composite hypertrophy scores. The strongest predictor of the PRE to W3 composite hypertrophy score was PRE type II fCSA values **(A)**. The strongest predictor of the W3 to W6 composite hypertrophy score was the percent change in type I fiber myonuclear number (MN#) **(B)**. The strongest predictor of the PRE to W6 composite hypertrophy score was PRE type II fCSA values **(C)**.

As stated prior, our second layer of analysis included performing stepwise linear regression in order to ascertain significant predictors of hypertrophy (i.e., the prediction variable in each model was the composite mean percent change score of the four clustering variables displayed in Figure 1). Notably, the strongest predictors are reported for each level of time with individual associated R^2^ values, standardized beta coefficients, and *p*-values as (R^2^ = β = p = ). For the overall analysis (*n* = 30), PRE to W3 variables that qualified for the model included PRE type II fiber percentage (*r* = 0.488), PRE type I fiber percentage (*r* = -0.488), PRE type II fCSA (*r* = -0.480), PRE type I fCSA (*r* = -0.462), PRE ubiquitin-labeled protein levels (*r* = 0.330), PRE-W3 type I fiber myonuclear number percent change (*r* = 0.381), and PRE-W3 muscle pan AMPKα percent change (*r* = -0.345). The strongest positive predictors from PRE-W3 were: (a) PRE type II fiber percentage (*R*^2^ = 0.238, β = 0.488, *p* = 0.006) and (b) PRE-W3 type I fiber myonuclear number percent change (*R*^2^ = 0.144, β = 0.379, *p* = 0.018). The strongest negative predictors from PRE-W3 were: (a) PRE type I fiber percentage (*R*^2^ = 0.238, β = -0.488, *p* = 0.006), (b) PRE type II fCSA (*R*^2^ = 0.241, β = -0.491, *p* = 0.001), and (c) PRE-W3 muscle pan AMPK percent change (*R*^2^ = 0.102, β = -0.332, *p* = 0.019). W3 to W6 variables that qualified for the model were: PRE muscle p-mTOR (*r* = -0.327), W3–W6 type I fiber myonuclear number percent change (*r* = 0.412), W3–W6 type II fiber myonuclear number percent change (*r* = 0.387), W3–W6 serum cortisol percent change (*r* = 0.33), W3–W6 muscle p-p70s6k percent change (*r* = 0.473), W3–W6 ubiquitin-labeled protein levels percent change (*r* = 0.318), and W3–W6 muscle MGF mRNA percent change (*r* = 0.353). The only significant positive predictor from W3 to W6 was the W3–W6 percent change in p-p70s6k (*R*^2^ = 0.223, β = 0.473, *p* = 0.020); although W3–W6 type I fiber myonuclear percent change neared significance (*p* = 0.067). No models including significantly strong negative predictors were surmised from W3 to W6 (*p* > 0.05). PRE-W6 variables that qualified for the model included PRE type II fiber percentage (*r* = 0.390), PRE type I fiber percentage (*r* = -0.390), PRE type II fCSA (*r* = -0.455), PRE type I fCSA (*r* = -0.431), and PRE muscle p-AMPKα (*r* = -0.301). The strongest positive predictor from PRE-W6 was PRE type II fiber percentage (*R*^2^ = 0.152, β = 0.390, *p* = 0.033). The strongest negative predictors were PRE type I fiber percentage (*R*^2^ = 0.160, β = -0.401, *p* = 0.024) and PRE type II fCSA (*R*^2^ = 0.207, β = -0.455, *p* = 0.019). The strongest predictors of the PRE to W3, W3 to W6, and PRE to W6 composite hypertrophy scores are illustrated in Figure 9 below.

## Discussion

This study continues to highlight differences in biomarkers that exist between low and high hypertrophic responders following weeks of high-volume resistance training. While our sample size is limited, this study is unique given that most of the previous reports in this area have examined untrained individuals, whereas this is only the second study to examine well-trained college-aged males. Additionally, we feel a strength of the current study is classifying clusters using a combination of hypertrophic indices.

The most provocative finding herein was that, relative to high responders, low responders exhibited greater PRE type II fCSA (+18%, *p* = 0.022) values. Our regression analysis also suggested that lower PRE type II fCSA values and a greater PRE type II fiber percentage were the strongest predictors of the PRE-W6 hypertrophic response. In agreement with past data in untrained individuals demonstrating that low responders possessed higher pre-training VL thickness values (Mobley et al., 2018), these current data also imply that well-trained high responders may possess more growth potential given that they began training with lower fCSA values relative to low responders. Admittedly, none of the assayed biomarkers provide clear insight as to why pre-training fCSA values were greater in low versus high responders or how PRE type II fiber percentage mechanistically relates to the hypertrophic response. Thus, future work in this area is needed to determine genes or biomarkers (e.g., connective tissue thickness, DNA methylation status, etc.) which may affect inherent muscle size and fiber type-specific responses, and whether they are altered between low versus high responders during periods of resistance training. Notwithstanding, this is the first study to our knowledge suggesting that pre-training fCSA values are greater in low versus high hypertrophic responders. Likewise, we are the first to suggest that pre-training muscle fiber type has predictive bearing on the hypertrophic response. Indeed, the fiber type data herein are not in agreement with prior research which has suggested that pre-training muscle fiber type percentages were similar between low and high response clusters (Bamman et al., 2007; Stec et al., 2016; Mobley et al., 2018). However, these past reports all examined untrained participants. Hence, the potential for predominant muscle fiber type to influence the hypertrophic response to high volume training in previously trained participants should be further explored.

Several studies have reported that, in previously untrained participants, ribosome biogenesis is greater in high versus low hypertrophic responders to resistance training (Figueiredo et al., 2015; Stec et al., 2016; Mobley et al., 2018). Our finding indicating that changes in ribosome biogenesis markers were similar between high and low responders which were previously trained, however, suggests that this process may be less critical in stimulating muscle hypertrophy over a 6-week training period.

Satellite cell-mediated myonuclear accretion seemingly occurs during longer-term periods of resistance exercise training, and data from Bamman’s and Phillips’ laboratories suggests that increases in satellite cell proliferation in response to one bout or weeks of resistance training are greater in high versus low responders (Petrella et al., 2008; Bellamy et al., 2014). In contrast, we recently reported that training-induced increases in satellite cell number and increases in types I and II fiber myonuclear number were similar between high versus low responders following 12 weeks of resistance exercise training (Mobley et al., 2018). While we did not assess satellite cell counts in the current study, no significant main effects or interactions were observed for either type I or type II myonuclear number per fiber which indicates that satellite-cell mediated myonuclear accretion may not differentiate skeletal muscle hypertrophy in previously trained college-aged males. However, it is interesting that the PRE-W3 percent change in type I fiber myonuclear number was a significant predictor of the percent change in the composite hypertrophy score. Hence, our regression results indicate that satellite cell-mediated type I fiber myonuclear addition could be involved with hypertrophy to a certain degree.

Human and rodent studies suggest the magnitude increase of mTORC1 signaling markers (e.g., p-p70s6k and p-4EBP1) following a resistance exercise bout are predictive of longer-term skeletal muscle hypertrophy (Baar and Esser, 1999; Terzis et al., 2008; Mitchell et al., 2014; Damas et al., 2016). While the W3–W6 percent change in p-p70s6k was a significant predictor of the W3–W6 change in the composite hypertrophy score, we observed no between-cluster differences in phosphorylated mTOR, p70s6k, 4EBP1, or AMPKα levels. These data suggest that mTORc1 signaling may not be critical in differentiating the hypertrophic response in previously-trained individuals, although these results should be interpreted with caution given that the W3 and W6 sampling time points were 24 h following a prior exercise bout. Alternatively stated, researchers typically assay these markers within the first 1–6 h following exercise given that these post-exercise time points yield robust changes (Farnfield et al., 2009; Haun et al., 2017), although there is data to suggest that p-mTOR (Ser2448), p-p70s6k (Thr289), and p-4EBP-1 (Thr37/46) are significantly elevated 24 h following a resistance exercise bout (Burd et al., 2011; Gundermann et al., 2014). Notwithstanding, it remains possible that these markers could have differed between clusters if biopsies were obtained in a more acute post-exercise time frame. Likewise, basal or post-exercise muscle protein and/or myofibrillar protein synthesis rates were not directly assessed in this investigation and could have differed between low and high responders.

We also sought to examine cluster differences in biomarkers related to muscle damage and proteolysis. Herein, we observed no between-cluster differences in serum CK activity levels, muscle 20S proteasome activity levels, or muscle ubiquinated protein levels. Likewise, our regression models indicated that none of these biomarkers significantly explained the variance in the composite hypertrophy score. These data agree with our prior data suggesting that neither muscle 20S proteasome activity nor MuRF-1 protein content differ prior to or following 12 weeks of resistance training in previously untrained males (Mobley et al., 2018). However, as with the lack of protein synthesis data herein, these data are limited in that we did not directly assess whether protein breakdown rates differed between clusters.

Androgen receptors operate as transcription factors to alter the mRNA expression of hundreds to thousands of genes (Jiang et al., 2009). A high level of enthusiasm exists regarding the hypertrophic effects of AR signaling given that the administration of anabolic steroids increases satellite cell proliferation (Sinha-Hikim et al., 2002; Sinha-Hikim et al., 2003) and MPS (Griggs et al., 1989; Ferrando et al., 1998). Two studies have reported that changes in skeletal muscle AR protein content correlate with increases in skeletal muscle hypertrophy. Ahtiainen et al. (2011) reported skeletal muscle AR protein increases correlated with fCSA and lean body mass increases in younger and older men following 21 weeks of resistance training. Mitchell et al. (2013) subsequently reported increases in muscle AR protein content correlate with muscle hypertrophy following 16 weeks of resistance training. More recently, Phillips’ laboratory reported that AR protein content was greater prior to and following 16 weeks of resistance training in previously trained high versus low responders (Morton et al., 2018). However, we recently reported that 12 weeks of resistance training downregulated AR content in high and low responders who were previously untrained (Mobley et al., 2018), and suggested this was a negative feedback phenomena regarding resistance training and AR protein expression. Given that the paper by Morton et al. (2018) was published during the writing of this manuscript, we decided to perform a *post hoc* analysis on muscle AR levels in only the HIGH and LOW clusters. In the current study we observed no between cluster effect or interaction for serum testosterone or AR protein content. Our findings are in partial agreement with other literature (Mobley et al., 2018; Morton et al., 2018) in that there was no training effect, between-cluster effect, or interaction regarding serum total testosterone levels. However, it is difficult to reconcile why our AR protein findings differ from Morton et al. (2018), and we took extra precaution to ensure that our antibody was valid using a positive control LNCaP lysate. Thus, more research is needed in determining if muscle AR protein content does separate high versus low hypertrophic responders.

We recently proposed high responders may possess a greater mitochondrial volume given that muscle anabolism requires high amounts of cellular energy for protein turnover (Roberts et al., 2018a). Likewise, we recently reported that high responders that were previously untrained possessed greater muscle citrate synthase activity levels than low responders (Roberts et al., 2018b). However, the current citrate synthase activity data suggest that mitochondrial content is similar in both clusters, and similarly decreases in both clusters with training. Notwithstanding, while these data suggest mitochondrial volume decrements occur in high and low responders that were previously trained, it does not rule out the potential that the low or high response clusters experienced alterations in mitochondrial function (e.g., states 3 and 4 respiration levels, complex activity levels, etc.).

It should be noted that back squat training volume did not differ between response clusters, and this finding is also in agreement with past data we collected in previously untrained college-aged male low versus high responder cohorts (Mobley et al., 2018). Additionally, self-reported macronutrient intakes did not differ between clusters, and our laboratory as well as Bamman’s laboratory have reported similar findings (Thalacker-Mercer et al., 2009; Mobley et al., 2018). Finally, while the number of participants in the LOW and HIGH clusters did not significantly or practically differ with regard to protein supplement consumption reported by Haun et al. (2018b), this does not rule out the potential that consuming large amounts of supplemental whey protein was primarily responsible for the hypertrophic response in the three HIGH responders assigned to this group.

### Experimental Limitations

There are limitations to this study. First, the original intent of this project was to utilize high volume resistance training to enhance hypertrophy. As such, we did not obtain a post-intervention strength metric. Thus, it is currently unclear as to whether pre- to post-changes in lower body strength differed between the HIGH and LOW clusters herein. A second limitation of this study is the lack of Pax7 staining for histologically detecting satellite cell changes with training between clusters. While this staining would have been informative, we chose to allocate our resources toward analyzing other biomarkers and depended solely upon detecting changes in myonuclear number through DAPI staining in order to make inferences regarding satellite cell-mediated fusion events. However, we cannot rule out the potential that the HIGH and LOW clusters herein contained different satellite cell numbers prior to or after the training intervention. Another histology limitation herein is the lack of discrimination between type IIa and type IIx fibers. While Bamman’s laboratory has reported that no baseline differences in types IIa and IIx fiber percentage exist between low and high hypertrophic responders (Bamman et al., 2007), this does not to rule out the possibility that differences existed in the currently analyzed clusters. Another limitation is that blood and biopsy sampling at the week 3 and week 6 time points occurred 24 h following the last training bout due to study logistics. This may have had an impact on some of the assayed biomarkers (e.g., serum CK and mRNA/rRNA expression patterns) and unfortunately is an unresolved limitation. Finally, like other similar studies examining hypertrophic responders and non-responders to resistance training (Davidsen et al., 2011; Stec et al., 2016; Morton et al., 2018), it is notable that this study was also limited in scope regarding n-size. In this regard, meta-analytical interrogations which combine all of these studies will likely be needed to obtain enough power in order to decipher which biomarkers differ between clusters.

## Conclusion

This study continues to delineate biomarkers that exist between low versus high responders to resistance training, but is unique in that it is only the second study to date that has examined subjects that were well-trained prior to engaging in the training protocol. As stated in a recent perspective on the topic (Roberts et al., 2018a), research identifying intrinsic factors that regulate differential hypertrophic responses to resistance exercise training will generate future research which examines if these factors can be modulated by altering extrinsic variables such as nutrition, exercise dosing, or recovery strategies. Furthermore, these series studies will ultimately improve our understanding of factors that optimize resistance exercise training adaptations, and such research will likely be useful for individuals seeking to apply this knowledge in a practical setting.

## Data Availability

All datasets generated for this study are included in the manuscript and/or the Supplementary Files.

## Author Contributions

CH and MDR were primarily responsible for the current research question, statistical analyses, and writing of the manuscript. CV critically assisted with all aspects of execution and analysis. MDR was the principal investigator of the laboratory where the work for this study was performed. All other co-authors assisted in multiple aspects of data collection as well as the preparation of the manuscript.

## Conflict of Interest Statement

JM is the Executive Director of Research and Education of Impedimed, Inc. who has extensive expertise in body composition assessment. He provided critical considerations for body composition assessments and data interpretation, but his involvement did not influence study results. The remaining authors declare that the research was conducted in the absence of any commercial or financial relationships that could be construed as a potential conflict of interest.

## Supplementary Material

FILE S1 Raw data from this study.
